# Practicing Apothecaries, Percentage on Prescriptions

**Published:** 1848-02

**Authors:** 


*Practicing Apothecaries, Percentage on Prescriptions.*—At the last meeting of the College of Physicians of Philadelphia, a committee, previously appointed to consider the subject of the practice pursued by apothecaries of prescribing for diseases and accidents, reported as follows:—

"The Committee having given the subject due consideration, are of opinion that it is in every way worthy of the attention of the College, and that much good may be anticipated from the action of the body in reference to it. The evil is one that in this community has long existed; its origin being propably due to the belief of the public, that those dealing in drugs, must be fully acquainted with their application to the cure of diseases; it being taken for granted that discrimination of the latter is an easy matter. The fact, too, that throughout a large portion of our country, physicians are in the habit of retailing their own medicines, and consequently, when they prescribe for a malady, of furnishing the remedies they direct; together with the vulgar notion, that each individual understands best his own constitution, have tended, in no small degree, to render the custom so common that it is by many thought to be perfectly right. Your committee, however, believe, that several of the apothecaries of this city have long been opposed to a practice for which they felt themselves unfitted, and continue it, to a limited extent, only from the force of circumstances—the competition at present existing in the business, compelling them to advise the use of various remedies, when consulted, in order to prevent the transfer of their customers to others, not in any degree beter qualified, and certainly less scrupulous.

Your committee, therefore, think that the action of the College in discountenancing the practice, will have a most beneficial effect; strengthening the opinions of those among the apothecaries who are now opposed to it, and deterring others from its continuance.

By limiting the duties of the apothecary to the compounding of medicines and the sale of articles called for by their customers, it is believed the public will be pretty well protected from a recurrence of such accidents as were reported to the college at its last meeting. The means of thus limiting the duties of the apothecaries would seem, it is true, not very easy to command, but as the compounding of physicians prescriptions is admitted by all in the business to be an object worth attending to, and as they gain nothing by. prescribing themselves, the considerations of self-interest will probably effect, to a certain extent at least, the desired end, even should higher motives be disregarded. If the College, therefore, were made acquainted with the views of apothecaries in regard to this matter, there would certainly be but little difficulty in inducing, by our co-operation, a large portion of them to adopt the views of the profession generally in relation to it.

Your committee, therefnre, recommend that the following circular be addressed to each apothecary in the city proper and southern districts."

[We omit the circular, the object of which is to ask the views and concurrence of the apothecaries. The report embraces another topic as follows:—]

As connected with the present subject, the committee would call the attention of the College, to the fact of certain physicians holding an interest in apothecary shops, or if not actually principals or partners, receiving a percentage on every precription sent to the store.

They beg leave, therefore, to offer the following resolutions:—

1st. *Resolved,* That in the opinion of the College, the duties of prescribing and compounding medicines in this city should be kept perfectly distinct, and that any connection or moneyed interest in apothecary stores, on the part of physicians, is unprofessional, and therefore, to be reprobated.

2d. *Resolved.* That the College of Physicians will hereafter deem those apothecaries who continue to prescribe for diseases as unworthy of the support of its Fellows.

3d. *Resolved,* That the Fellows of this College be recommended to exert themselves individually and collectively to abolish the practice.

